# Response to Multiple Stressors: Enhanced Tolerance of *Neoseiulus barkeri* Hughes (Acari: Phytoseiidae) to Heat and Desiccation Stress through Acclimation

**DOI:** 10.3390/insects10120449

**Published:** 2019-12-13

**Authors:** Ji Huang, Ming-Xiu Liu, Yang Zhang, Zai-Yin Kuang, Wei Li, Chang-Bin Ge, Ya-Ying Li, Huai Liu

**Affiliations:** 1 Key Laboratory of Entomology and Pest Control Engineering, College of Plant Protection, Southwest University, Chongqing 400716, China; huangjirh@163.com (J.H.); lmx0044@126.com (M.-X.L.); zzfddzy@163.com (Y.Z.); zaiyinkuang@163.com (Z.-Y.K.); liwei17749967405@163.com (W.L.); liyaying@swu.edu.cn (Y.-Y.L.); 2 Luohe Academy of Agricultural Sciences, Luohe 450002, China

**Keywords:** multiple stressors, predatory mite, desiccation, high temperature, acclimation

## Abstract

Organisms are always confronted with multiple stressors simultaneously. Combinations of stressors, rather than single stressor, may be more appropriate in evaluating the stress they experience. *N. barkeri* is one of predatory mite species that are commercialized for controlling spider mites. However, their biological control efficiency was often reduced because of high temperature and desiccation in summer. To understand how to improve the tolerance of *N. barkeri* to combined heat and desiccation stress, we pre-exposed the adult female of *N. barkeri* to high temperature, desiccation and high temperature × desiccation stress for acclimation. After proper recovery time, mites were subjected to high temperature × desiccation stress again to detect the acclimation effects. The results are as follows: (1) No decrease in mortality rate were observed under high temperature × desiccation stress after heat acclimation. Instead, it increased significantly with acclimation temperature and time. (2) Dehydration acclimation both at 25 °C and high temperatures reduced mortality rate under high temperature × desiccation stress. Mortality rate was only significantly correlated with the amount of water loss, but not with temperature or water loss rate in acclimation, suggesting the increased tolerance is related to dehydration stress rather than heat stress. Among all acclimations, chronic dehydration at 25 °C, 50% relative humidity were the most effective treatment. This study indicated dehydration acclimation is effective to enhance tolerance of *N. barkeri* to combined heat and desiccation stress, which can improve the efficiency of biological control under multiple stressors.

## 1. Introduction

Terrestrial arthropods and other organisms are confronted with various stressors, such as heat, cold, insecticide, and UV-B [1,2,3,4]. Considering that different stressors would affect organisms interactively in various possible ways, combinations of stressors rather than single stressor, may be more important in predicting how organisms react to environment [5].

The interactive effect of two stressors have been extensively studied in marine ecosystems. Conceptually, two stressors can interactively affect organisms in an additive, antagonistic or synergistic way [6]. Based on meta-analysis of multi-stressor effects on organisms in marine ecosystem, synergistic effect appears to be the most common in multi-stressor studies [7]. This may be the same case with terrestrial arthropods since synergistic interactions of multi-stressors were also common among insects [8,9,10]. This indicates that the effects of multi-stressors are more complicated than the simply additive influence.

Predatory mites are excellent natural enemies that are commercially produced to control phytophagous mites, thrips, and other small pests. However, their biological control outcomes are influenced by abiotic factors, such as high temperature and desiccation [11,12]. In summer, predatory mites experience high temperature and desiccation simultaneously, as relative humidity (RH) at leaf surface decreases when temperature increases. Such micro-environmental conditions can reduce survival and reproduction of predatory mites [3,13,14], especially when the plants are drought stressed [15] or the weather is windy [16]. Although there was no report about the interactive effects between high temperature and desiccation, it was observed that the survival of predatory mites was severely and adversely impacted when high temperature and desiccation occurred simultaneously compared when to these factors occurred alone. With the increase of temperature and vapor pressure deficit (VPD, corresponding to “drying power”), survival time of predatory mites decreased but mortality increased, especially for the more vulnerable larval stage [3,14,17]. Consequently, under hot and dry conditions, the biological control efficacy of predatory mites against pest mites is often insufficient [11,12].

Acclimation to this stress for a short-term or long-term period is one of the strategies to enhance resistance or tolerance of natural enemies to stressful condition. Short-term acclimation was proved to be effective in improving resistance or tolerance of organisms to cold [18], heat [19], and desiccation stress [20]. Long-term acclimation has been used to screen for stress resistant strains, such as insecticide-resistant [21], heat-adapted predatory mites [22] and desiccation-resistant fruit fly [23]. However, the previous research only focused on a one-stressor situation. Although many researches have reported cross-tolerance between multiple stressors [24], little is known about how to improve resistance or tolerance of arthropods to combined stress through acclimation.

In this study, the predatory mite *Neoseiulus barkeri* Hughes (Acari: Phytoseiidae) was used as it is commercially produced and extensively used in controlling spider mites. To explore which acclimation method can improve the tolerance of *N. barkeri* to the combined stress of heat and desiccation, we subjected female mites to heat stress, desiccation stress, and heat × desiccation stress respectively during acclimation. After acclimation, we compared mortality of female mites under heat × desiccation condition. In this study, we found desiccation and heat × desiccation acclimation reduced mortality of predatory mites. To find out the factor that contribute to reduced mortality, the relationships between mortality rate and water loss amount, water loss rate, and temperature were explored.

## 2. Materials and Methods

### 2.1. Mite Rearing and Preparation

The predatory mite, *N. barkeri*, was purchased from the Institute of Plant Protection, Chinese Academy of Agricultural Sciences, Beijing, China. The rearing methods were similar to those described by Zhang [25]. Briefly, wheat bran with suitable water content was placed at the bottom of translucent plastic rearing boxes. Then enough flour mite *Aleuroglyphus ovatus* and *N. barkeri* were separately added into these boxes that are placed in programmable temperature controllers (Ningbo Southeast Instrument Co. Ltd., RDN-300B-4, Ningbo, China) with temperature at 25 ± 1 °C, 70%–80% RH, and L:D = 14:10. Homogeneous adult female mites were prepared by the methods described in Zhang’s study with some modifications [26]. Briefly, a plastic film with 10 cm diameter was placed on a round sponge that was soaked with distilled water in a Petri dish (15 cm in diameter). Mites were separated from wheat bran using a screen mesh, and then were transferred into a Petri dish (5 cm in diameter) which was placed on the plastic film (in the bigger Petri dish). Then female mites were picked out into another Petri dish (15 cm in diameter) with soaked sponge and plastic film. Some cowpea leaves were placed on plastic film to provide place for female mites to lay eggs. Eggs laid within 24 h were collected, and then transferred into a 4 L translucent plastic box with enough flour mites and wheat bran, and held at 25 °C, 70%–80% RH and L:D = 14:10 in programmable temperature controllers, the same condition with conventional rearing. Six days after egg collection (more than 90% mites developed into adult [27]), mites were screened out again as described before [26]. Ten homogeneous female adults were transferred into a small acrylic cage that was adapted from Williams et al. [28]. Generally, the cage was made by transparent acrylic (3 cm × 3 cm × 0.3 cm), with a central circular hole (1.6 cm diameter), covered on one side by a metal mesh (300 mesh number) and the other side with a piece of glass. Cages and mites were maintained at 25 °C and 100% RH in chambers for acclimation use.

### 2.2. Desiccation Acclimation

**Figure 1:**
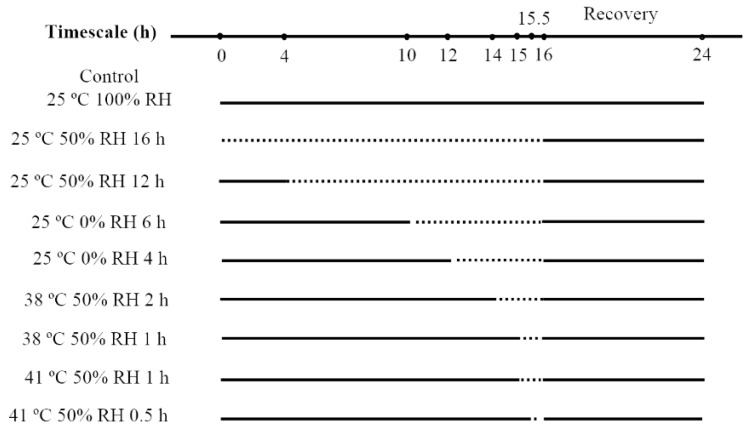
Process of desiccation acclimation of *N. barkeri*. The dash lines represent duration of desiccation acclimation, and the solid lines represent the duration under 25 °C, 100% relative humidity (RH).

At low humidity condition, dehydration occurs both at normal temperature (25 °C) and high temperatures (38 °C and 41 °C, corresponding to common high temperature and extreme high temperature in summer of Chongqing respectively [29]). To find out the optimal temperature for acclimation, we dehydrated the predatory mites at 25 °C, 38 °C, and 41 °C respectively. As atmosphere RH is always below 50% in a summer day during the hottest several hours [15,30], the humidity 50% was selected as the humidity in acclimation (We also chose 0% RH as acclimation humidity at 25 °C to test if it has the potential for mass use in desiccation acclimation, as this humidity is easy to generate and cost-saving). Due to water loss rate decreased in the following order: 41 °C, 50% RH > 38 °C, 50% RH > 25 °C, 0% RH > 25 °C, 50% RH, different acclimation periods were set to achieve a consistent water loss amount of mites in the above treatments. The pilot study showed mites began to die when they lost water more than 20% of their total weight (Figure S1). Thus two levels of water loss amount were set in acclimation. Level 1 (about 20% weight loss) includes the treatments: 25 °C, 50% RH for 16 h, 25 °C, 0% RH for 6 h, 38 °C, 50% RH for 2 h, 41 °C, 50% RH for 1 h. Level 2 (weight loss between 10–15%) includes the treatments: 25 °C, 50% RH for 12 h, 25 °C, 0% RH for 4 h, 38 °C, 50% RH for 1 h, 41 °C, 50% RH for 0.5 h (illustrated in Figure 1). Distilled water, saturated Mg(NO_3_)_2_ solution and anhydrous calcium chloride were filled in 4 L translucent plastic boxes to generate the humidity of 100%, 50%, and 0%, respectively [28,31]. These boxes were maintained in programmable temperature controllers with the temperature of 25 °C, 38 °C, and 41 °C. The set values and observed values of temperatures and humidities were shown in Table S1. Three acrylic cages with 10 mites per cage were set up for each treatment.

### 2.3. Heat Acclimation

**Figure 2:**
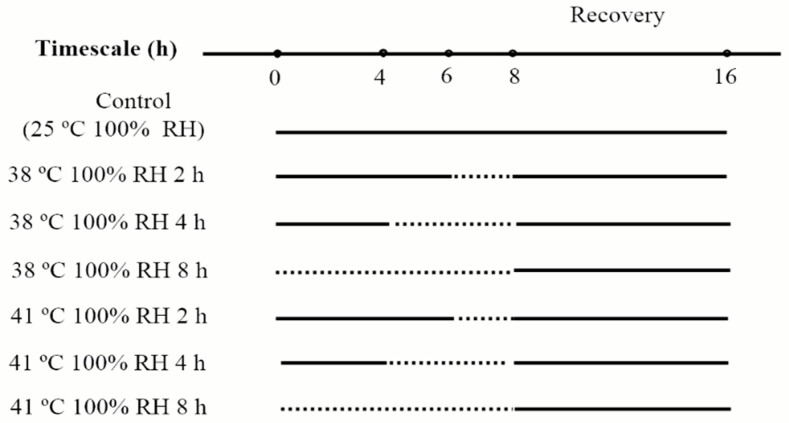
Process of heat acclimation of *N. barkeri*. The dash lines represent the duration of heat acclimation, and the solid lines represent the duration under 25 °C, 100% RH.

In heat acclimation, mites were exposed to the following acclimation treatments, with temperature regimes (38 °C and 41 °C) crossed with acclimation periods (2 h, 4 h, 8 h), resulting in six treatments. To avoid dehydration of mites, distilled water was put at the bottom of a 4 L plastic box to keep humidity in this box near saturation. These boxes were kept in chambers with the acclimation temperatures. For each treatment, four acrylic cages containing 40 mites were put on mesh supporter over distilled water in a plastic box. These boxes were kept in chambers (Ningbo Southeast Instrument Co. Ltd., RDN-300B-4, Ningbo, China) that generate the temperatures in acclimation. After heat acclimation, mites were kept at 25 °C, 100% RH for 8 h to recover (to keep recovery time the same with that in desiccation acclimation, for detailed process, see Figure 2). The control mites were kept at 25 °C, 100% RH without heat acclimation or recovery.

### 2.4. Mortality Tests

After acclimation, mites were kept at 25 °C, 100% for 8 h to recover. Eight hours was chosen as recovery time because female *N. barkeri* with different water loss levels could fully rehydrate at 25 °C, 100% RH for 6 h, then body weight keep stable after 6 h. Mites in control were kept at 25 °C, 100% RH without acclimation and recovery. After recovery, mites were exposed to the stress of 38 °C, 50% RH and 41 °C, 50% RH. The survival of mites was checked at 4 h and 6 h under 38 °C, 50% RH and at 2 h and 3 h under 41 °C, 50% RH. The observation time was set according to our pilot study exploring the survival rate of *N. barkeri* under different temperature and humidity combinations. Mites did not move a distance of 1 mm in 30 s after shaking the acrylic cage were considered dead. All treatments were repeated for three times.

### 2.5. Water Loss Test

To find out how desiccation acclimation contribute to the reduced mortality under heat and desiccation stress, we investigated water loss amount (WLA, % of total weight) and water loss rate (WLR, % of total weight per hour) in different treatments during dehydration acclimation. About 200–300 adult female mites (1–2 mg) with similar body size were transferred into a metal mesh cage (2 cm × 2 cm × 0.4 cm in size). These metal cages were made of aluminum to avoid water absorption, with a central circular hole (1.6 cm diameter), covered on one side by a metal mesh (300 mesh number), the other side by a piece of glass which was tied to cages by an iron wire. Mites and mesh cages were initially weighted to the nearest 1 μg (*m*_0_) by sartorius MSA6.6S-OCE-DM microbalance before treatment. Then metal mesh cages containing mites were held at the same temperature and humidity combinations with that in desiccation acclimation (that is 25 °C, 50% RH, 25 °C, 0% RH, 38 °C, 50% RH and 41 °C, 50% RH, see “Section 2.2”). Mites were reweighted at 0.5 h and 1 h at 41 °C, 50% RH; at 1 h and 2 h at 38 °C, 50% RH; at 4 h and 6 h at 25 °C, 0% RH and at 12 h and 16 h at 25 °C, 50% RH to get weight *m_i_*. After weighting, mites were swept out, and the cage was weighted again to get its clean weight *m*_1_. Each treatment has three replicates. WLA and WLR were calculated as follows (modified from Gefen [32]):

(1) $$\mathrm{WLA}=\frac{m_{0\;}-m_{i}}{m_{0\;}-m_{1}}$$

(2) $$\mathrm{WLR}=\frac{{(m}_{0}{-m}_{i})}{(m_{0}-m_{1})\;t}$$

where “*t*” represents the periods between *m_i_* and *m*_0_. WLR is calculated at “*t*” when about 20% weight loss was observed, to reduce difference in WLR caused by different WLA. The values of WLA and WLR were listed in Table S2.

### 2.6. Statistical Analysis

Prior to ANOVA, mortality rate was arcsine square-root transformed to meet the assumptions of normality and homogeneity of variance. Then the impact of acclimation temperature, acclimation duration and the interactive effect of temperature × duration on mortality under heat and desiccation stress after acclimation was analyzed by a two-way ANOVA, where mortality rate was set as responding variable and temperature and time as predicting variables. The difference in mortality rate between heat treatments was analyzed by a one-way ANOVA, and then followed by a least significant difference (LSD, *p* < 0.05) test. Mortality rate after dehydration acclimation was subjected to a one-way ANOVA followed also by LSD (*p* < 0.05) test to detect the effect of dehydration acclimation on survival of mites under heat and desiccation stress.

Linear regression analysis was conducted to explore relationships of mortality rate with temperature, WLR and WLA during acclimation. ANOVA analyses were performed using SPSS statistics 20 (IBM Statistics, SPSS 2011) for Windows. Linear regression analyses were performed with Origin Pro 9.0 (Fang and Ye 2004).

Though difference in relative humidity between set values and measured values were found, e.g., measured humidity value was 4.7% higher than the set value 50% RH at 25 °C, 4.6% higher than 0% RH at 25 °C (Table S1), we used set values rather than measured values in this paper for convenience.

## 3. Results

### 3.1. Dehydration Acclimation on Mortality Rate

**Figure 3:**
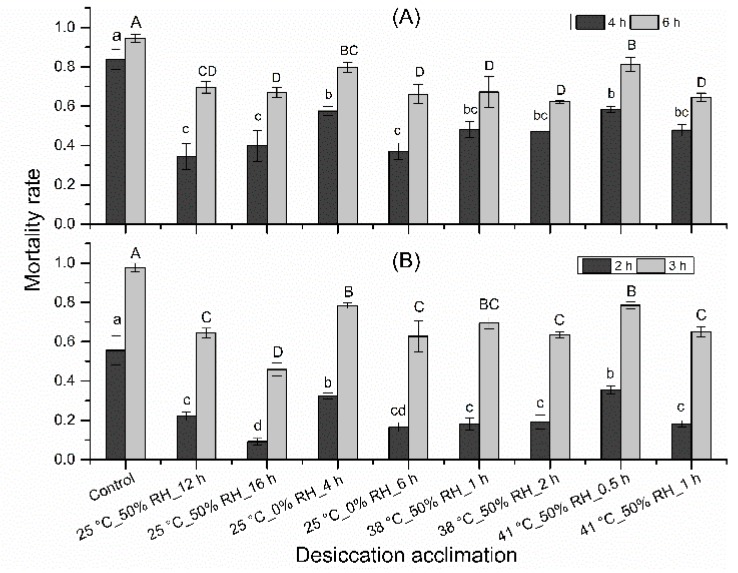
Effects of dehydration acclimation on the mortality of *N. barkeri* under heat and desiccation stress. (**A**) mortality at 4 h and 6 h under 38 °C, 50% RH for different acclimation regimes; (**B**) mortality at 2 h and 3 h under 41 °C, 50% RH for different acclimation regimes. The difference of mortality rate among all treatments and control was analyzed by a least significant difference (LSD, *p* < 0.05) test. Different lower letters on the top of each bar indicate significant difference in mortality rate at 38 °C, 50% RH for 4 h or at 41 °C, 50% RH for 2 h (*p* < 0.05). Different capital letters on the top of each bar indicate significantly difference in mortality rate at 38 °C, 50% RH for 6 h or at 41 °C, 50% RH for 3 h (*p* < 0.05).

All dehydration treatments led to significant decrease in mortality rate (Figure 3; stressed at 38 °C, 50% RH for 4 h: *F* = 10.61; df = 8,26; *p* < 0.001; stressed at 38 °C, 50% RH for 6 h: *F* = 9.90; df = 8,26; *p* < 0.001; stressed at 41 °C, 50% RH for 2 h: *F* = 17.75; df = 8,26; *p* < 0.001; stressed at 41 °C, 50% RH for 3 h: *F* = 19.75; df = 8,26; *p* < 0.001).

When mites were stressed at 38 °C, 50% RH for 4 h, 83.7% mites died in control, the mortality rate of all acclimation treatments ranged from 34.3% to 58.4%, increasing in the following order: 25 °C, 50% RH for 12 h < 25 °C, 0% RH for 6 h < 25 °C, 50% RH for 16 h < 38 °C, 50% RH for 2 h < 41 °C, 50% RH for 1 h < 38 °C, 50% RH for 1 h < 25 °C, 0% RH for 4 h < 41 °C, 50% RH for 0.5 h. When stressed at 38 °C, 50% RH for 6 h, 94.5% mites died in control, the mortality rate of all acclimation treatments ranged from 62.2% to 81.3%, increasing in the following order: 38 °C, 50% RH for 2 h < 41 °C, 50% RH for 1 h < 25 °C, 0% RH for 6 h < 25 °C, 50% RH for 16 h < 38 °C, 50% RH for 1 h < 25 °C, 50% RH for 12 h < 25 °C, 0% RH for 4 h < 41 °C, 50% RH for 0.5 h. When mites were stressed at 41 °C, 50% RH for 2 h, 55.6% mites died in control, the mortality rate of all acclimation treatments ranged from 9.2% to 35.4%, increasing in the following order: 25 °C, 50% RH for 16 h < 25 °C, 0% RH for 6 h < 38 °C, 50% RH for 1 h < 41 °C, 50% RH for 1 h < 38 °C, 50% RH for 2 h < 25 °C, 50% RH for 12 h < 25 °C, 0% RH for 4 h < 41 °C, 50% RH for 0.5 h. When mites were stressed at 41 °C, 50% RH for 3 h, 97.8% mites died in control, the mortality rate of all acclimation treatments ranged from 45.8% to 78.5%, increasing in the following order: 25 °C, 50% RH for 16 h < 25 °C, 0% RH for 6 h < 38 °C, 50% RH for 2 h < 25 °C, 50% RH for 12 h < 41 °C, 50% RH for 1 h < 38 °C, 50% RH for 1 h < 25 °C, 0% RH for 4 h < 41 °C, 50% RH for 0.5 h. Totally, as acclimation time increased, the mortality rate decreased at heat and desiccation stress, except acclimation under 25 °C, 50% RH.

### 3.2. Heat Acclimation on Mortality Rate

**Figure 4:**
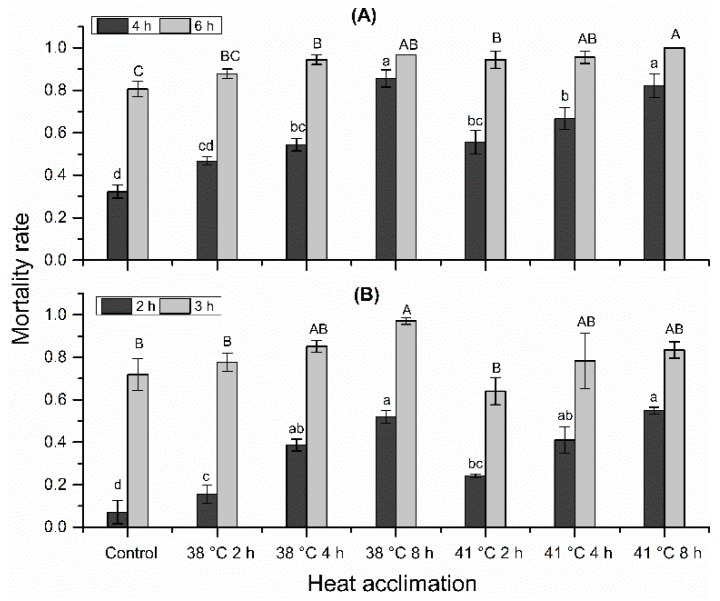
Effects of heat acclimation on the mortality of *N. barkeri* under heat and desiccation stress. (**A**) mortality at 4 h and 6 h under 38 °C, 50% RH for different acclimation regimes; (**B**) mortality at 2 h and 3 h under 41 °C, 50% RH for different acclimation regimes. The difference of mortality rate among all treatments and control was analyzed by a least significant difference (LSD, *p* < 0.05) test. Different lower letters on the top of each bar indicate significant difference in mortality rate at 38 °C, 50% RH for 4 h or at 41 °C, 50% RH for 2 h (*p* < 0.05). Different capital letters on the top of each bar indicate significantly difference in mortality rate at 38 °C, 50% RH for 6 h or at 41 °C, 50% RH for 3 h (*p* < 0.05).

Mortality rates under 38 °C, 50% RH and 41 °C, 50% RH were not reduced by heat acclimation. Instead, mortality rate increased significantly with acclimation temperature (Figure 4; stressed at 38 °C, 50% RH for 4 h: *F* = 40.28; df = 2, 14; *p* < 0.001; stressed at 38 °C, 50% RH for 6 h: *F* = 15.79; df = 2, 14; *p* < 0.001; stressed at 41 °C, 50% RH for 2 h: *F* = 33.93; df = 2, 14; *p* < 0.001; stressed at 41 °C, 50% RH for 3 h: *F* = 3.99; df = 2, 14; *p* = 0.042) and acclimation time (stressed at 38 °C, 50% RH for 4 h: *F* = 27.46; df = 2,14; *p* < 0.001; stressed at 38 °C, 50% RH for 6 h: *F* = 4.42; df = 2,14; *p* = 0.032; stressed at 41 °C, 50% RH for 2 h: *F* = 17.95; df = 2,14; *p* < 0.01; stressed at 41 °C, 50% RH for 3 h: *F* = 4.11; df = 2,14; *p* = 0.039).

When mites were stressed at 38 °C, 50% RH for 4 h, 32.3% mites died in the control, the mortality rate of all acclimation treatments ranged from 46.7% to 85.6%, increasing in the following order: 38 °C for 2 h < 38 °C for 4 h < 41 °C for 2 h < 41 °C for 4 h < 41 °C for 8 h < 38 °C for 8 h. When stressed at 38 °C, 50% RH for 6 h, 80.6% mites died in the control, the mortality rate of all acclimation treatments ranged from 87.8% to 100%, increasing in the following order: 38 °C for 2 h < 38 °C for 4 h < 41 °C for 2 h < 41 °C for 4 h < 38 °C for 8 h < 41 °C for 8 h. When stressed at 41 °C, 50% RH for 2 h, 7.1% mites died in control, the mortality rate of all acclimation treatments ranged from 15.6% to 54.9%, increasing in the following order: 38 °C for 2 h < 41 °C for 2 h < 38 °C for 4 h < 41 °C for 4 h < 38 °C for 8 h < 41 °C for 8 h. When stressed at 41 °C, 50% RH for 3 h, 71.9% mites died in control, the mortality rate of all acclimation treatments ranged from 77.6% to 97.1%, increasing in the following order: 41 °C for 2 h <38 °C for 2 h < 41 °C for 4 h < 41 °C for 8 h < 38 °C for 4 h < 38 °C for 8 h. Unexpectedly, mites acclimated at 41 °C had lower mortality rate than the counterparts acclimated at 38 °C when stressed at 41 °C, 50% RH for 3 h. This phenomenon was analyzed in Discussion.

### 3.3. Regression Analysis

**Figure 5:**
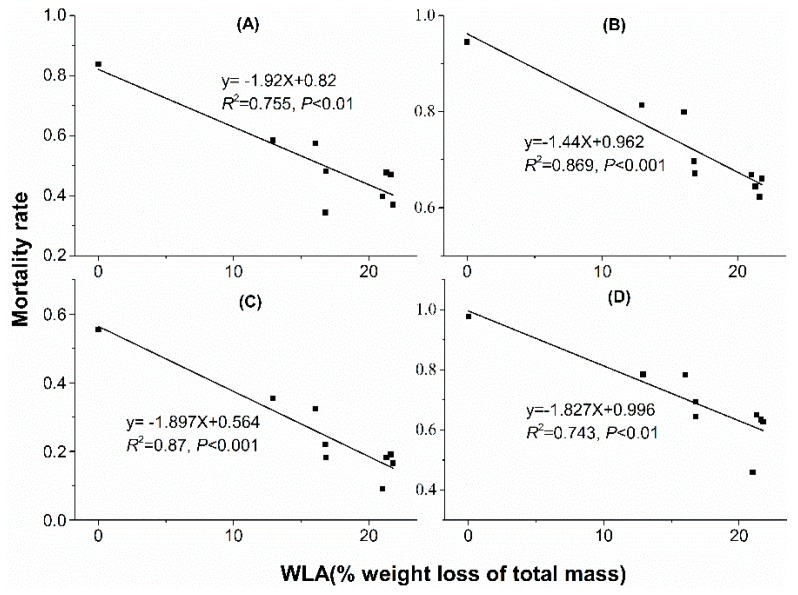
Linear regression between mortality rate and water loss amount in dehydration acclimation of *N. barkeri*. (**A**) mortality rate under 38 °C, 50% RH for 4 h; (**B**) mortality rate under 38 °C, 50% RH for 6 h; (**C**) mortality rate under 41 °C, 50% RH for 2 h; (**D**) mortality rate under 41 °C, 50% RH for 3 h. WLA: water loss amount.

**Figure 6:**
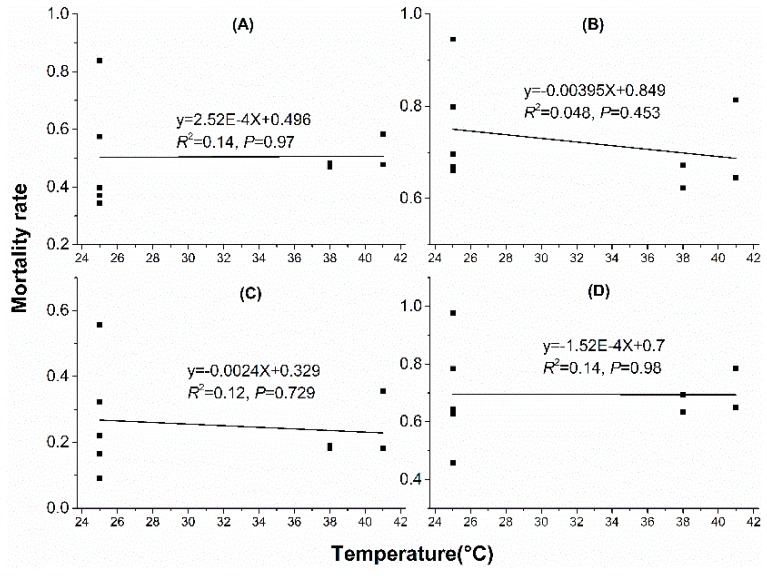
Linear regression between mortality rate and temperature in dehydration acclimation of *N. barkeri*. (**A**) mortality rate under 38 °C, 50% RH for 4 h; (**B**) mortality rate under 38 °C, 50% RH for 6 h; (**C**) mortality rate under 41 °C, 50% RH for 2 h; (**D**) mortality rate under 41 °C, 50% RH for 3 h.

**Figure 7:**
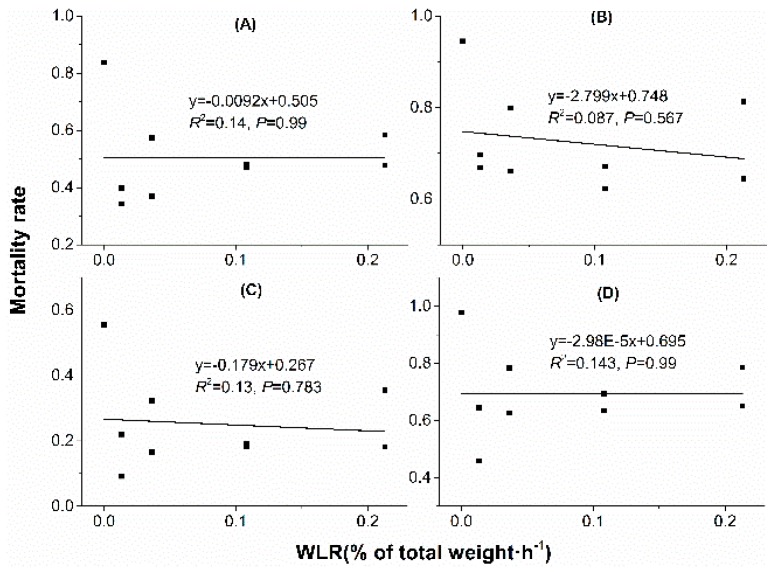
Linear regression between mortality rate and water loss rate (WLR) in dehydration acclimation of *N. barkeri*. (**A**) mortality rate under 38 °C, 50% RH for 4 h; (**B**) mortality rate under 38 °C, 50% RH for 6 h; (**C**) mortality rate under 41 °C, 50% RH for 2 h; (**D**) mortality rate under 41 °C, 50% RH for 3 h.

Linear regression analyses showed that water loss amount in acclimation significantly correlated with mortality rate. With water loss amount increased in acclimation, mortality rate decreased when mites were stressed at 38 °C, 50% RH for 4 h (Figure 5A), 38 °C, 50% RH for 6 h (Figure 5B), 41 °C, 50% RH for 2 h (Figure 5C) and 41 °C, 50% RH for 3 h (Figure 5D). However, no significant correlation was found between mortality rate and water loss rate (WLR) or temperature (Figure 6 and Figure 7).

## 4. Discussion

### 4.1. The Effect of Dehydration Acclimation on Mortality Rate

In this study, dehydration acclimation increased survival rate of *N. barkeri* under heat and desiccation stress, which is consistent with previous studies showing that dehydration pre-treatment always lead to enhanced desiccation tolerance and survival time in the subsequent desiccation stress in flies [23], collembolan [33], and nematode [34]. Thus we proved that under the combined stress of heat and desiccation, enhanced desiccation tolerance may be critical for increasing the survival rate of *N. barkeri* at the tested temperatures and humidities.

We found the mortality rates under high temperature and desiccation stress negatively correlated with WLA as mites had undergone dehydration acclimation. Similar findings were also reported in other studies. Within non-lethal acclimation time during dehydration, resistance of *Drosophila melanogaster* to the following desiccation stress was improved with the increase of acclimation time [23]. *Drosophila simulans* acclimated at lower humidity levels had higher resistance to desiccation than that acclimated at higher humidities both at 31 °C and 35 °C [35]. Both higher temperature and lower humidity mean higher drying power (corresponding to higher VPD), which always leads to higher WLA with the same stress duration. Previous studies have found evidence that higher WLA in acclimation can result in increased desiccation resistance by accumulating higher concentration of metabolites, such as glucose and trehalose [33,36]. Whether the concentration of metabolites of mites is higher after dehydration acclimation in this study is unknown and needs further exploration.

The lowest mortality rate was observed in mites acclimated at 25 °C, 50% RH (Figure 3) when water loss amount was at the same level in all treatments. This may be because chronic dehydration acclimation can trigger higher resistance to subsequent desiccation stress than acute dehydration dose. This has also been reported in other studies. For example, when the larvae of *Belgica antarctica* dehydrated too quickly (stressed at 0% RH), they become less resistant to desiccation than their counterpart stressed at 75% or 98% RH [36]. In collembolan *Cryptopygus antarcticus*, these dehydrated at 98.2% RH was more desiccation tolerant than those dehydrated at 75% RH with similar WLA [33]. Both these two insects mentioned above accumulated significantly higher concentrations of metabolites, such as glucose, trehalose, and glycerol, when desiccated with slower WLR with similar WLA [33,36]. This may partly explain the difference of desiccation resistance between chronic and acute dehydration acclimation.

The mechanism of enhanced survival under heat and desiccation stress is still largely unknown. Many studies found enhanced desiccation resistance of insects after short-term or long-term desiccation acclimation. Insects acquire enhanced desiccation resistance or tolerance mainly through three ways: (1) Higher body water content after acclimation, (2) having lower water loss rate by reducing respiratory water loss or cuticular permeability, and (3) more tolerant of water loss before death [37,38]. The last one was more prevailing in anhydrobiotic organisms [37,39,40], and may be not observed in this mite. However, which way or ways account for enhanced survival rate under heat and desiccation stress in *N. barkeri* needs further study.

### 4.2. Effect of Heat Acclimation on Mortality Rate

In this study, we did not observe improved tolerance to combined stress of heat and desiccation after heat acclimation. Instead, mortality rate increased significantly with temperature and time of acclimation (Figure 4). The heat-induced desiccation tolerance seems to be context dependent. In some cases, no obvious effect of heat hardening on desiccation resistance was found, such as for the fruit fly *D. simulans* [35] and chrysomelid *Chirodica chalcoptera* [41]. While higher temperature induced greater desiccation resistance in *Eldana saccharina* (Lepidoptera: Pyralidae) [42], collembolan *Pogonognathellus flavescens* [43] and flesh flies *Zaprionus indianus* [24]. On the contrary, some studies found lower desiccation resistance after higher temperature acclimation. For instance, *D. nepalensis* reared at 15 °C had greater desiccation resistance compared with that reared at 25 °C [44]. Heat hardening at 36 °C and high humidity reduced desiccation resistance of adult *Drosophila melanogaster* compared with the non-hardened controls [45]. This is in agreement with our findings in this study. Metabolites, such as glucose, trehalose, and glycerol, are crucial sugars and polyols protecting arthropods from desiccation damage. Higher temperatures will increase metabolic rates in ectotherms, causing a faster depletion of storage metabolites [44,46,47]. Besides, coping with desiccation stress is an energy consuming process that requires more carbohydrates than usual [48]. Thus, heat induced faster depletion of metabolites may account for higher mortality rate of *N. barkeri* under heat and desiccation stress.

Unexpectedly, mites acclimated at 41 °C had lower mortality rate than the counterparts acclimated at 38 °C when stressed at 41 °C, 50% RH for 3 h, which cannot be explained by the depletion of metabolites. Zhang has found much higher expression level of heat shock protein (Hsp 70) genes in female mites of *N. barkeri* after 1 h exposure to 42 °C than those exposed to 38 °C [49]. Thus, we suspect that female mites acclimated at 41 °C accumulated more Hsp 70 than those acclimated at 38 °C, which may partly explain the lower mortality rate of mites acclimated at 41 °C when stressed at 41 °C, 50% RH for 3 h [50].

### 4.3. Acclimation Under Combined Stress

Arthropods have to deal with various stressors simultaneously. It is difficult to predict how these stressors would impact on arthropods, since they can interact with each other in various ways. Knowledge on how multiple stressors interactively affect organisms and how organisms respond to them is crucial toward a comprehensive understanding of the relationship between organisms and the environment. In this study, we attempted to enhance the tolerance of *N. barkeri* to combined heat and desiccation stress through different acclimation strategies. We showed desiccation acclimation rather than heat acclimation contributed to enhanced tolerance. This may indicate that at the tested temperatures and humidities, the mortality of *N. barkeri* was mainly led by dehydration instead of heat stress, and the survival rate of *N. barkeri* was positively related to enhanced desiccation tolerance. Thus we may get a new perspective of understanding how organisms interact with multiple environmental stressors through different acclimation strategies.

Improving the biological control efficiency of predatory mites under stress condition is of huge economic significance, especially under multiple stressors. In this study, previous acclimation to dehydration stress led to lower mortality rate of mites when they were subsequently exposed to heat and desiccation stress. This was most obvious in mites pre-dehydrated at rearing temperature (25 °C). By using desiccant (e.g., anhydrous calcium chloride), low humidity condition was easy to generate at rearing temperature. Thus, desiccation acclimation is feasible for enhancing tolerance of predatory mites to heat and desiccating stress in mass production, increasing the achievement of biological control to spider mites, especially in hot and arid areas.

## 5. Conclusions

Our results showed that desiccation acclimation, both at 25 °C and high temperatures, significantly enhanced the tolerance of females *N. barkeri* to combined heat and desiccation stress (38 °C, 50% RH and 41 °C, 50% RH), However, high temperature acclimation increased their sensitivity to combined stress. Among all desiccation acclimation treatments, the mortality rates were only significantly correlated with WLA, but not with WLR or temperature. Therefore, our study suggested that desiccation acclimation rather than heat acclimation can enhance the tolerance of adult female of *N. barkeri* to combined heat and desiccation stress.

## Supplementary Materials

The following are available online at https://www.mdpi.com/2075-4450/10/12/449/s1. Figure S1. Mortality and water loss amount of *Neoseiulus barkeri* female under 38 °C, 50% RH. Table S1: Set values (mean ± se) and observed values (mean ± se) of temperature and humidity in this study, Table S2: Water loss amount (Mean ± se) and water loss rate (Mean ± se) of *Neoseiulus barkeri* in different acclimation treatment.
