# Supplementary material for: Response to Multiple Stressors: Enhanced Tolerance of Neoseiulus barkeri Hughes (Acari: Phytoseiidae) to Heat and Desiccation Stress through Acclimation
Source: Insects. 2019 Dec 13;10(12):449. doi: 10.3390/insects10120449 (PMC6956224; doi:10.3390/insects10120449)
Supplement: Supplementary file 1 [file insects-10-00449-s001.pdf]

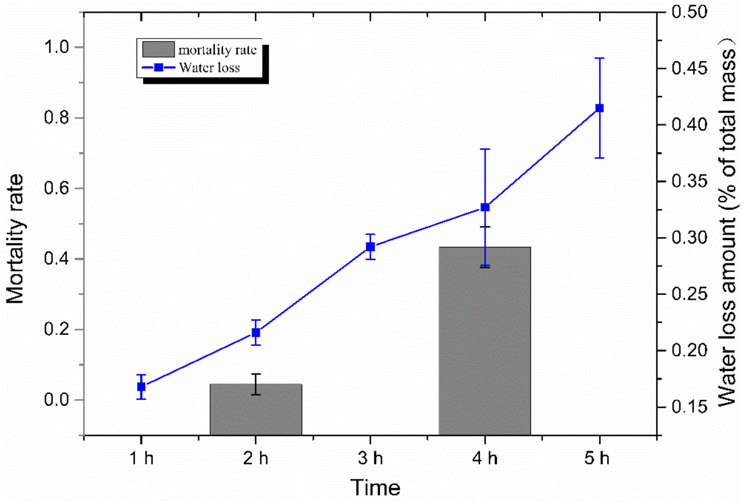

Fig. S1. Mortality and water loss amount of *Neoseiulus barkeri* female under 38 °C, 50% RH. The methods used to test mortality rate and water loss amount was the same described in main text of "Materials and Methods" without acclimation.

Figure 2. Mortality of *N. barkeri* females under 41 °C, 50% RH both grouped and individually. Group: 10 mites/cell, 3 cells/treatment; Individual: 1 mite/cell, 10 cells/ treatment. All treatments repeat 3 times.

Table 1. Set values (mean ±se) and observed values (mean ± se) of temperature and humidity in this study.

**Temperature (°C)**		Relative humidity (%)		Solution
**Set values**	**Observed values**	Set values	Observed values	
		100	99.8 ± 0.1	Distilled water
25	25.0 ± 0.1	50	54.7 ± 0.1	$Mg(NO_3)_2$
		0	4.6 ± 0.0	$CaCl_2$
38	38.1 ± 0.1	50	49.4 ± 0.1	$Mg(NO_3)_2$
41	41.1 ± 0.1	50	48.8 ± 0.0	$Mg(NO_3)_2$

Temperature and humidity of the chambers were checked 48 h after boxes had been put to set temperatures for equilibrium

Table 2. Water loss amount (Mean ± se) and water loss rate (Mean ± se) of *Neoseiulus barkeri* in different acclimation treatment.

**Treatment**	**WLA (% of total weight)**	**WLR (% of total weight per hour)**

Control (25 °C 100% RH)	0	0
25 °C 50% RH 12 h	16.78 ± 0.90	1.40 ± 0.07
25 °C 50% RH 16 h	21.0 ± 1.08	1.31 ± 0.07
25 °C 0% RH 4 h	16.03 ± 1.61	4.01 ± 0.40
25 °C 0% RH 6 h	21.79 ± 2.68	3.63 ± 0.45
38 °C 50% RH 1 h	16.82 ± 1.86	11.21 ± 1.24
38 °C 50% RH 2 h	21.61 ± 1.57	10.80 ± 0.78
41 °C 50% RH 0.5 h	12.91 ± 0.42	25.82 ± 0.83
41 °C 50% RH 1 h	21.30 ± 0.58	21.30 ± 0.58
